# Predicting tumour content of liquid biopsies from cell-free DNA

**DOI:** 10.1186/s12859-023-05478-8

**Published:** 2023-09-30

**Authors:** Mathias Cardner, Francesco Marass, Erika Gedvilaite, Julie L. Yang, Dana W. Y. Tsui, Niko Beerenwinkel

**Affiliations:** 1 Department of Biosystems Science and Engineering, ETH Zurich, 4058 Basel, Switzerland; 2 SIB Swiss Institute of Bioinformatics, 4058 Basel, Switzerland; 3 Department of Pathology, Memorial Sloan Kettering Cancer Center, New York, NY USA; 4 Epigenetics Research Center, Memorial Sloan Kettering Cancer Center, New York, NY USA; 5 Present Address: PetDx, Inc, La Jolla, USA

**Keywords:** Liquid biopsy, Tumour content, Cell-free DNA, Circulating tumour DNA, Fragmentomics

## Abstract

**Background:**

Liquid biopsy is a minimally-invasive method of sampling bodily fluids, capable of revealing evidence of cancer. The distribution of cell-free DNA (cfDNA) fragment lengths has been shown to differ between healthy subjects and cancer patients, whereby the distributional shift correlates with the sample’s tumour content. These fragmentomic data have not yet been utilised to directly quantify the proportion of tumour-derived cfDNA in a liquid biopsy.

**Results:**

We used statistical learning to predict tumour content from Fourier and wavelet transforms of cfDNA length distributions in samples from 118 cancer patients. The model was validated on an independent dilution series of patient plasma.

**Conclusions:**

This proof of concept suggests that our fragmentomic methodology could be useful for predicting tumour content in liquid biopsies.

## Introduction

For the past decade, the primary goal in the analysis of plasma cfDNA has been the detection of tumour material. To this end, extremely sensitive assays have been developed to classify samples as healthy or with cancer [1–6], with boundaries being pushed towards earlier detection and detection of minimal residual disease after treatment [7, 8]. Beyond binary classification, another question of interest is how to monitor disease burden non-invasively, for example to assess a patient’s response to treatment. A cfDNA proxy for disease burden is plasma tumour content, i.e., the proportion of DNA originating from the tumour.

Monitoring strategies require tumour-specific features that reflect tumour burden quantitatively. The main genomic approaches that have been used until now have focussed on mutations or copy-number alterations (CNAs). Mutation-based monitoring requires sufficiently high sequencing depth to accurately determine mutations and their allele frequency [6]. To properly estimate tumour burden, one needs to account the CNAs and the clonal structure of the tumour, thus estimating tumour burden from copy-number-corrected clonal mutations in the sample [9]. More affordable in terms of sequencing volume is the CNA-based approach, which leverages shallow whole-genome sequencing (sWGS) of cfDNA to identify copy-number events and estimate the proportion of tumour DNA in the sample [10]. The drawback of this strategy is that the information in coverage-based data alone is insufficient for uniquely determining tumour burden, and multiple equally good fits to the data are possible [11, see also our Additional file 1]. As such, there is no guarantee that these estimates will be correct. We avoid the drawbacks of these approaches and propose a third option.

Following observations that the distribution of cfDNA lengths differs between fragments of tumour and non-tumour origin [12], we reasoned that informative features specific to the tumour could be derived directly from it. The fragment length distribution displays multiple modes corresponding to nucleosomes, and an oscillatory pattern on the short side of each of these modes, corresponding to full twists of nucleosome-bound DNA. Compared to healthy controls, samples with higher tumour content contain a greater proportion of short fragments, and may also have more pronounced oscillations for fragment sizes within one nucleosome [13–15]. After extracting features from this distribution, our fragmentomic approach to predicting systemic tumour burden is framed as a regression problem. In the following, we show a proof of concept of our methodology and apply it to a data set of 118 cfDNA samples, followed by a validation of its predictions in a dilution series which was not used during training.

## Results

We analysed data published by Tsui et al. [16] consisting of plasma samples from 118 patients with stage IV cancer. The cancer types, and the numbers of patients afflicted, were: bladder (45), prostate (35), breast (15), germ cell (11), lung (11), melanoma (1). For a comprehensive characterisation, please refer to Tsui et al. [16]. In addition, we analysed a serial dilution of a cancer patient’s plasma mixed with healthy control plasma. Samples were profiled using both sWGS and deep panel sequencing from the same library preparation. Because supervised learning requires labelled data, we derived mutation and CNA information from the panel-sequencing data and used it to manually curate tumour content labels.

The 118 patients were split into two cohorts based on whether the panel-sequencing data contained the information necessary for direct estimation of tumour content (please refer to the *Methods* section for details and a schematic). For 41 patients, designated cohort A, there was at least one point mutation in copy-number neutral regions, enabling direct estimation of tumour content from the variant allele frequencies (Additional file 1: Figure S1). We considered these estimates to be the most reliable. For the remaining 77 patients, designated cohort B, the panel-sequencing data did not allow for direct estimation of tumour content. To gauge the tumour content of these samples, we relied on estimates by the tool *ichorCNA* [10]. For comparison, in cohort A the tumour-content estimates based on panel-sequencing data correlated well with those given by *ichorCNA* (Pearson’s $r=77\%$; Additional file 1: Figure S2). In light of this correlation, we reasoned that cohort B could be used to shortlist informative features, even though we preferred to use cohort A to calibrate the final model. We therefore decided to use the *ichorCNA*-based estimates as a surrogate during feature selection, but we used only the direct estimates during parameter tuning.

**Fig. 1:**
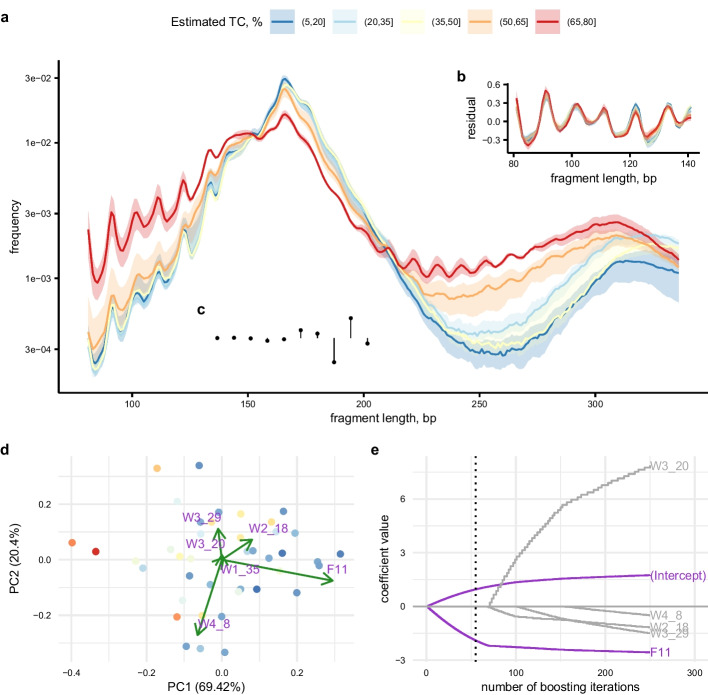
Cohort A data and feature extraction. **a** Fragment length distributions of cfDNA from 41 patient samples grouped by tumour content estimated from panel-sequencing data. Per group, the median profile is shown banded by the interquartile range, with the *y*-axis on a logarithmic scale. **b** Residuals of second-degree polynomial regression models fitted in the range 81–141 bp. The discrete Fourier transform was applied to each set of residuals. **c** The Daubechies wavelet filter was used to extract features in a data-driven fashion across the full range of 81–336 bp. **d** Biplot of PCA based on the features chosen separately by stability selection (based on cohort B, not shown). Variable loadings are illustrated by green arrows with purple labels where ‘Wx_y’ denotes the wavelet coefficient on scale *x* at location *y*, and ‘Fz’ refers to the absolute value of the *z*th Fourier coefficient. **e** Coefficient paths of beta boosting applied to the selected features. The dotted vertical line indicates the optimal number of iterations, as determined by cross-validation

The cfDNA fragment length distributions exhibited characteristics previously described in the literature, namely an oscillatory pattern leading up to the first peak, centred at 166 bp, and an overabundance of short fragments in tumour-derived cfDNA [13, 14, 17]. In line with previous reports, we observed an enrichment of short fragments in samples with high tumour content (Fig. 1a). This enrichment was most evident for nucleosome-protected DNA, i.e., the ranges leading up to the first and second nucleosomal peaks.

We consistently visualised and modelled the cfDNA fragment length distributions on a logarithmic scale. To extract features from the oscillatory pattern in the 81–141 bp range, we applied the discrete Fourier transform to residuals of a second-degree polynomial fit to each fragment length distribution in that interval (Fig. 1b). Additionally, to capture transient signals at different scales and locations, we employed a discrete wavelet transform of the entire range 81–336 bp (Fig. 1c).

Based on the 71 samples in cohort B with non-zero estimates of tumour content, we used beta-regression boosting [18] coupled with complementary-pairs stability selection [19, 20] to identify Fourier and wavelet coefficients informative for the prediction of tumour content. In a principal component analysis based on the selected features, 69% of the variance in cohort A was explained by the first component, for which the absolute value of the eleventh Fourier coefficient $F_{11}$ (please see *Methods* for its definition) had a particularly high loading (Fig. 1d). Indeed, after fitting a beta-boosting model to cohort A and tuning it using cross-validation, only $|F_{11}|$ remained in the final model (Fig. 1e). At a granular level, it appeared to be mainly the real part of $F_{11}$ which correlated with tumour content (Additional file 1: Figure S3).

**Fig. 2:**
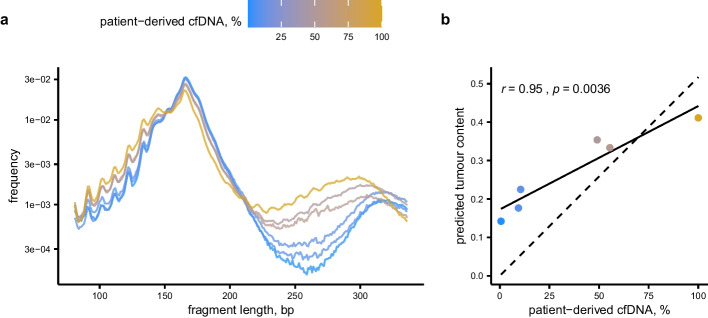
Assessment of model predictions in a held-out test data set. **a** Fragment length distributions of cfDNA in a serial dilution of patient plasma into control plasma. **b** Predicted tumour content in the dilution series. The solid line corresponds to simple linear regression, showing that the model’s predictions correlate well with the concentration of patient-derived cfDNA. The dashed line is constrained to cross the origin, highlighting that our trained model overestimates tumour content in the low range

To test the predictive performance of the model in an independent data set, we turned to the dilution experiment in which one patient’s plasma was mixed with control plasma at six distinct concentrations. Thus, the test set, albeit derived from a single patient, is about 15% of the size of the labelled training set ($n=41$). An additional benefit of using the dilution experiment as a test set was that the concentration of patient-derived plasma was known, meaning that we expected to see a linear trend between concentration levels and predicted tumour content. Implicitly, this also evaluated the method used to directly estimate the tumour content from panel-sequencing data. The cfDNA fragment length distributions from the dilution series were consistent with the training data, in that increasing concentrations of patient plasma showed an enrichment of fragments of length 81–141 bp as well as 220–300 bp (Fig. 2a).

The predicted tumour content correlated well with the concentrations of spiked-in cfDNA from a cancer patient, resulting in a coefficient of determination, $R^2$, of 0.90 (Fig. 2b). This validated both that the model generalised to unseen data, and that our estimates of tumour content in cohort A were adequate. However, the regression line does not intercept the *y*-axis at zero, suggesting that our model overestimated tumour content at lower dilutions. If constraining the regression line to intercept zero, the $R^2$ dropped to 0.83. This may be because the model was trained on samples with relatively high tumour content (the lower quartile being 14%), without control samples with exactly zero tumour content.

## Discussion

Our study should be considered a proof of concept, and longitudinal data from interventional studies in larger cohorts would be needed to develop the methodology and evaluate its utility in clinical practice. Before we discuss limitations and conclude with the potential implications of our study, a number of methodological points warrant consideration. Curation of the copy number and mutation data is a crucial step as it provides the labels used for training. All somatic mutations in copy-number neutral regions should be analysed, regardless of their relevance in the tumour, in order to have sufficient signal for mutation clustering. Alternatively, clonal reconstruction methods can be applied, though the only result of interest here is the tumour content of the sample [9].

A limitation of our study is the size of the data set. Having unbiased sWGS data as well as ground truth labels for the same samples requires an experimental design that has thus far not been the norm. The establishment of larger cohorts, where both deep and shallow sequencing are performed from the same library preparation, should be considered. As more data become available, our analysis may also be performed by tumour type, in case there are systematic differences in fragment length distribution between cancer types. Here, we did not model effects of such clinical features, since our data set was small. Instead, we attempted to predict tumour content as is, regardless of possible confounding factors. Our approach should also be evaluated on samples from patients with earlier stages of cancer, and in combination with methods based on point mutations and CNAs. The indication that our model overestimated the tumour content at low dilutions could be a consequence of having trained it on samples with high tumour content, taken from patients with stage IV cancer. We expect the predictive performance to improve and become less biased as more samples become available for feature selection and training. Additionally, longitudinal data from interventional studies would be needed to assess the ability of our methodology to monitor systemic tumour burden during treatment, and how it compares with estimates derived using imaging data.

Different protocols for DNA extraction, library preparation, sequencing, and read processing affect the fragment size profile [21, 22]. Aggregations of data must therefore eliminate such effects, for example through alignment of a large number of control samples, before our predictive model can be applied.

## Conclusions

We presented a proof-of-concept computational method for predicting tumour content in plasma samples by leveraging Fourier and wavelet transforms of the fragment length distribution of cfDNA. Strikingly, our analysis singled out as most informative the coefficient $F_{11}$ of the discrete Fourier transform applied to residuals in the range 81–141 bp. The trained model performed well in a small independent test set, though it appeared to underestimate tumour content at low dilutions. Further studies would be needed to evaluate the clinical utility of the method. Our approach relied on deep as well as shallow sequencing from matched library preparations to train the predictive model. However, subsequent applications to predict tumour content would only require inexpensive sWGS data, in principle enabling accessible prediction of tumour burden during the course of disease.

Fragmentomics is a rich source of biological information, whose potential is only beginning to be discovered and exploited. Fragmentomics-only methods have been proposed to detect cancer and understand the biological processes underlying cfDNA release and its degradation [3, 7, 23, 24]. With our method, we propose another clinical use for this data type. As more data are collected and the model refined, we envision this as a promising and inexpensive strategy for non-invasive monitoring of cancer patients.

## Methods

To estimate tumour content based on panel-sequencing data, we considered single-nucleotide variants (SNVs) only in copy-number neutral regions (excluding any copy-number neutral loss of heterozygosity). Assuming each point mutation to be heterozygous, the proportion of the tumour harbouring each mutation is twice the mutation allele frequency. Mutations found in all clones cluster at the largest allele frequency, and the tumour content of the sample can be obtained by doubling this number. We performed this analysis for each patient, clustering mutations by their allele frequency with a Chinese restaurant process from the R package *cloe* [25]. The maximal cluster was used to obtain the tumour content estimate, unless this cluster was supported only by non-annotated SNVs, in which case it was disregarded in favour of the second-maximal cluster.

We used the discrete Fourier transform to extract features from the oscillatory pattern at 81–141 bp after regressing out a second-degree polynomial fit to the fragment length distribution on a log scale. That is, for each sample we considered the proportion $p_j$ of cfDNA fragments of length $j\in [81,141]$, and fitted the model $\log (p_j)=\beta _0+\beta _1j+\beta _2j^2+\varepsilon _j$ by ordinary least squares to yield the residuals $(\hat{\varepsilon }_n)_{n=0}^{60}$ where $n=j-81$. Then we computed the Fourier coefficients $F_k = \sum _{n=0}^{60} \hat{\varepsilon }_n e^{-2\pi ikn/61}$ for $k\in [0,60]$, and used their absolute values $|F_k|$ as features. Since the residuals are centred at zero, $F_0=0$. Note that for $m=61-k$, the complex conjugate $\bar{F}_m=F_k$, meaning that $|F_m|=|F_k|$. Thus we considered only $|F_k|$ where $k\in [1,30]$ for downstream analysis. All computations were performed using the *lm* and *fft* functions in R [26]. Motivated by the oscillation period of 10 bp, we chose the Daubechies wavelet of length 10 as the basis function for a discrete wavelet transform in the range 81–336 bp. The wavelet transform extracted features from the fragment length distribution by computing weighted averages at different scales and locations. Wavelet coefficients were computed with the package *wavelets* [27].

**Fig. 3:**
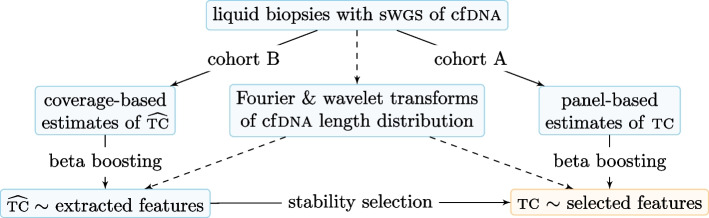
Modelling strategy in the training set. The final model is represented in the bottom-right corner

We used beta regression [28] to model tumour content as a response variable in (0, 1), with Fourier and wavelet coefficients as features (Fig. 3). Patients were split into two cohorts based on whether their plasma samples had sufficient panel-sequencing data to estimate tumour content from SNVs in copy-number neutral regions (cohort A) or not (cohort B). For cohort B, we relied on estimates of tumour content generated by *ichorCNA* [10]. As the number of samples was smaller than the number of features, we used boosting to fit high-dimensional models via the package *betaboost* [18]. The package *stabs* [29] was used in cohort B to perform complementary-pairs stability selection of informative features, with at most one expected false positive. The final beta-regression boosting model based on the informative features was trained on cohort A, and the number of boosting iterations was tuned using cross-validation by 25-fold bootstrap. For each fold and boosting iteration, the cross-validated empirical risk was computed based on the out-of-sample observations. The risk scores were then averaged across folds, and the number of boosting iterations with the lowest mean risk was chosen as optimal.

Finally, we applied the trained model to an independent test set, consisting of a dilution series. The predicted tumour content was compared with the dilution, and the concordance was assessed using linear regression with or without an intercept term.

### Supplementary Information

**Additional file 1.** Supplementary text and figures.

**Additional file 2.** A knitted R Markdown document for reproducing the main results.

## Data Availability

The data sets supporting the conclusions of this article are available in the GitHub repository https://github.com/TsuiLab/sWGS. These were derived from sequencing data previously published by Tsui et al. [16] and corresponding mutation calls available on cBioPortal [30]. The *R Markdown* code for reproducing the main results is available in the Additional file 2.
