# Supplementary material for: Predicting tumour content of liquid biopsies from cell-free DNA
Source: BMC Bioinformatics. 2023 Sep 30;24:368. doi: 10.1186/s12859-023-05478-8 (PMC10543881; doi:10.1186/s12859-023-05478-8)
Supplement: Supplementary file 1 — Additional file 1. Supplementary text and figures. [file 12859_2023_5478_MOESM1_ESM.pdf]

Supplementary Material

Cardner, Marass et al., *Predicting tumour content of liquid biopsies from cell-free DNA*

Unidentifiability of CNAs and tumour content

Given a patient sample, the tumour content $t \in (0, 1)$, estimated from the fragment length distribution, is the proportion of DNA coming from tumour cells out of all cfDNA in the sample. We partition the genome into B bins and denote by r_i the number of observed reads in bin $i \in \{1, 2, \ldots, B\} =: [B]$. Each bin i has a mixture CN $\operatorname{mix}(t, c_i)$ that produced the data r_i, and is defined as $\operatorname{mix}(t, c_i) := tc_i + (1 - t) \cdot 2$, where c_i is the unknown tumour CN. We model the latter as a real number rather than an integer because it is an average across tumour clones with different sizes and potentially different copy numbers. Finally, we define the haploid sequencing mass $\alpha > 0$ to model sequencing depth: α reflects the number of reads that a single copy of the genome is expected to yield, ignoring for simplicity covariates such as GC-content and mappability.

It is natural to expect the number r_i of reads in bin $i \in [B]$ to scale linearly with the mixture copy number $\operatorname{mix}(t, c_i)$. Given the above definition of the haploid sequencing mass α, we suppose that $\mu_i := \mathbb{E}r_i = \alpha \operatorname{mix}(t, c_i)$. Furthermore, the expected total number of mapped reads $M := \mathbb{E} \sum_i r_i = \sum_i \mu_i = \alpha \sum_i \operatorname{mix}(t, c_i)$, which yields $\alpha = M / \sum_i \operatorname{mix}(t, c_i)$. The denominator simplifies to $2(1 - t)B + t \sum_i c_i$. In particular, for a copy-number neutral genome we get $\alpha = M/(2B)$, which tracks with intuition. However, for a given sequence $\boldsymbol{\mu}$, the copy-number profile $\boldsymbol{c}$ is not identifiable without fixing t. To see this, consider a copy number profile $\boldsymbol{c}$ and multiply each c_i by $\gamma > 0$ to yield $\mu_i = \alpha_\gamma \operatorname{mix}(t_\gamma, \gamma c_i)$. Solving $\alpha \operatorname{mix}(t, c_i) = \mu_i = \alpha_\gamma \operatorname{mix}(t_\gamma, \gamma c_i)$ for $t_\gamma \in (0, 1)$ yields

$$t_\gamma = \frac{t}{\gamma(1 - t) + t} \tag{1}$$

excepting $c_i = \sum_i c_i / B$ (the mean), in which case both sides of the equation are $1/B$ for all t and γ. In other words, if $(\boldsymbol{c}, t)$ is a valid fit to $\boldsymbol{\mu}$, then so too is $(\gamma \boldsymbol{c}, t_\gamma)$; we can accommodate any γ by calibrating t_γ accordingly. However, by estimating t from the fragment length distribution, which is independent of the read depth, one can remove one degree of freedom from the equation, thereby making the problem identifiable.

Moreover, given a sequence $\boldsymbol{\mu}$ of mean depths, there is a circular dependence between estimating the haploid sequencing mass α and simultaneously inferring the copy number profile $\boldsymbol{c}$. The circularity arises from estimating $\hat{\alpha} = M / \sum_i \operatorname{mix}(t, c_i)$ while solving $\mu_i = \hat{\alpha} \operatorname{mix}(t, c_i)$ for c_i. To avoid this problem, we can assume that the median m of μ reflects copy-number neutrality, and restrict our attention to the subset of bins $S = \{i \in [B] : \mu_i = m\}$. Then, following the reasoning above, we can proceed by setting $\hat{\alpha} = \sum_S \mu_i / (2|S|) = m/2$.

Figures

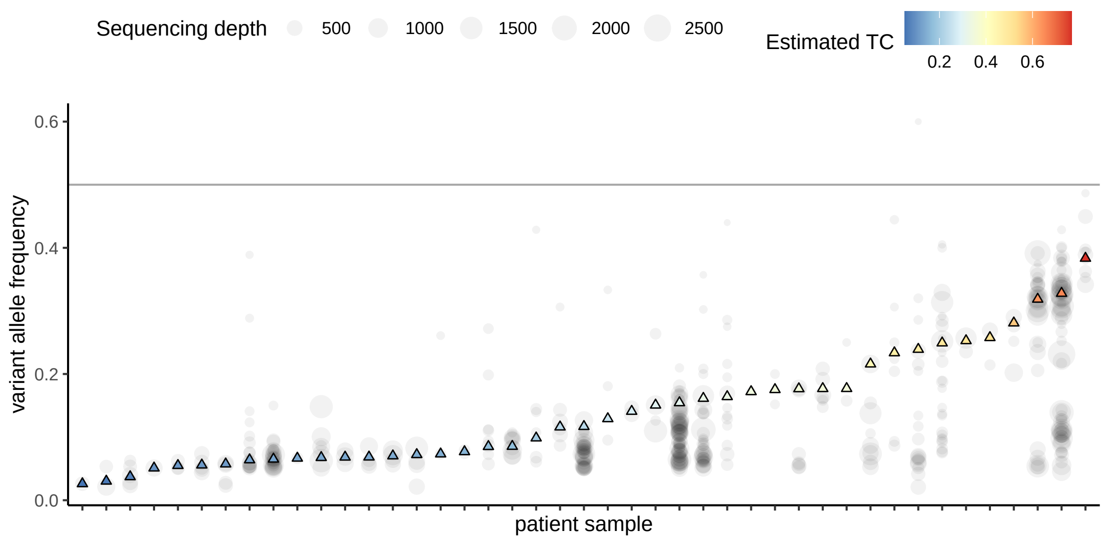

Figure S1: Allele frequencies of SNVs in copy-number neutral regions, based on panel sequencing of plasma samples from cohort A. Triangles show inferred cluster centres of clonal mutations.

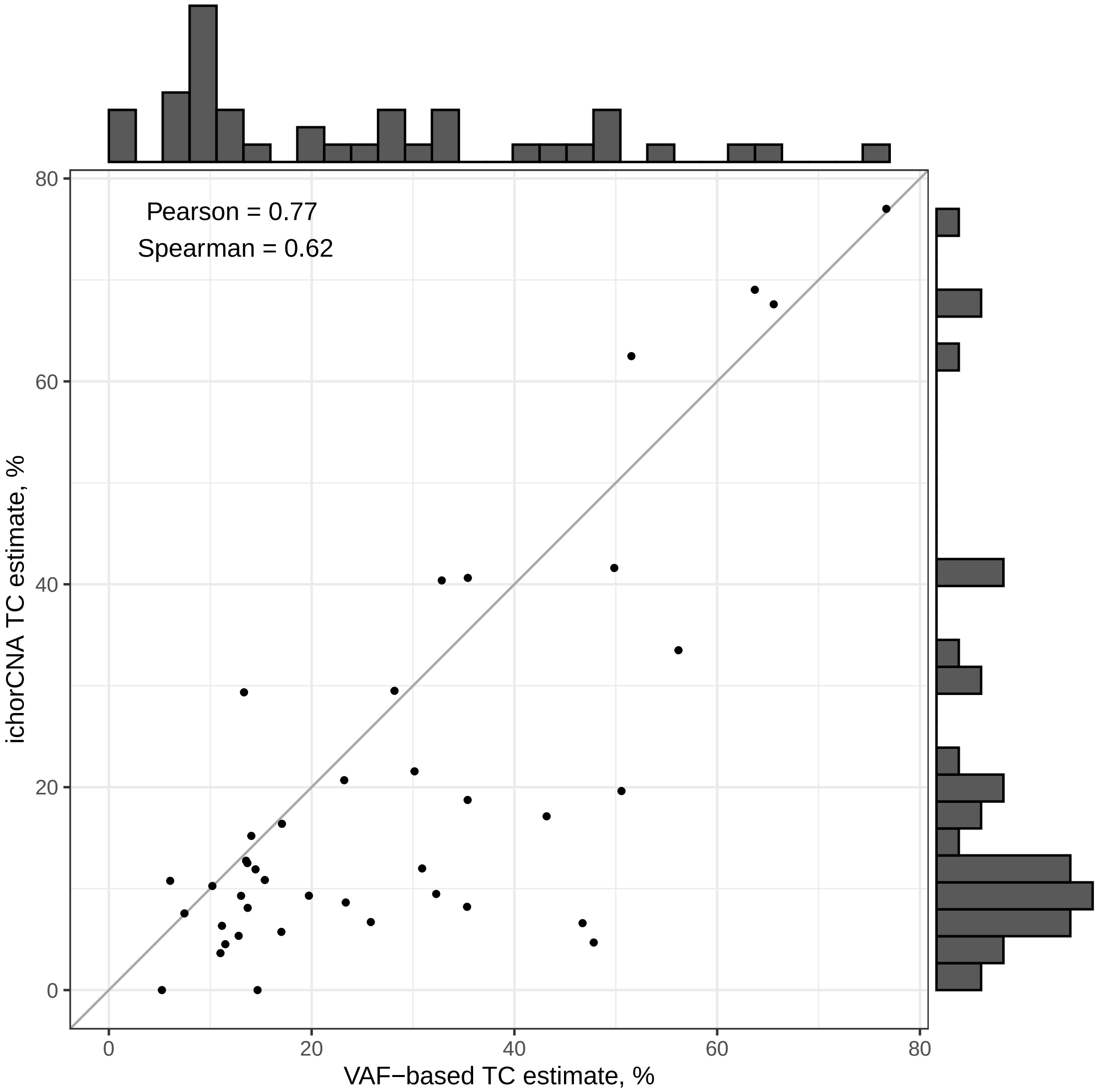

Figure S2: Tumour content in cohort A, as estimated by *ichorCNA* and the VAF-based procedure illustrated in Figure S1.

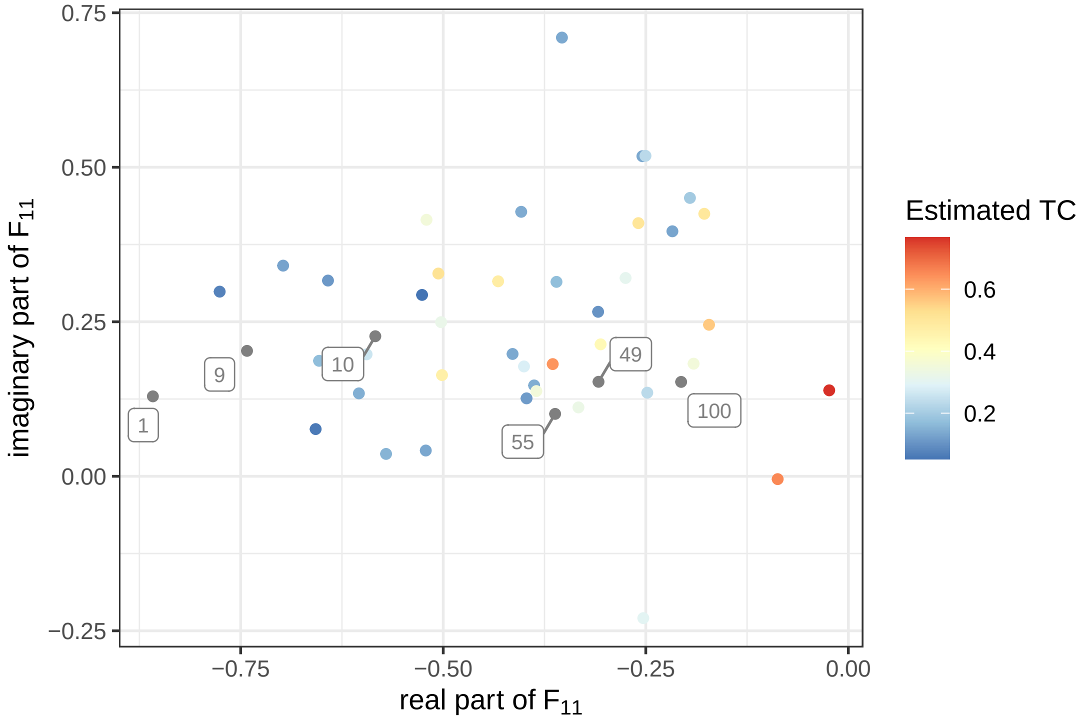

Figure S3: Real and imaginary parts of F_{11}, i.e., the eleventh coefficient of the discrete Fourier transform applied to residuals in the range 81–141 bp. The 41 patient samples with direct estimates of tumour content (TC) are coloured accordingly. The six samples from the dilution experiment are coloured grey and labelled by the percentage of patient-derived cfDNA. We observe that the real part (x-axis) appears to roughly order samples from low to high TC and dilution concentration, respectively.
