# Supplementary material for: Predicting tumour content of liquid biopsies from cell-free DNA
Source: BMC Bioinformatics. 2023 Sep 30;24:368. doi: 10.1186/s12859-023-05478-8 (PMC10543881; doi:10.1186/s12859-023-05478-8)
Supplement: Supplementary file 2 — Additional file 2. A knitted R Markdown document for reproducing the main results. [file 12859_2023_5478_MOESM2_ESM.pdf]

Predict tumour content

Beta boosting on Fourier and wavelet features

Mathias Cardner

8 August 2022

Here we apply discrete Fourier and wavelet transforms to the cfDNA fragment length distributions (FLDs). Then we use gradient boosting and stability selection on cohort B (ichorCNA labels) to identify features predictive of tumour content. The selected features are used to fit a boosting model on cohort A (CRP-derived labels).

Technical requirements

- This markdown requires that the GitHub repository https://github.com/TsuiLab/sWGS has been cloned into `data/` in the working directory.
- In case the `betaboost` package is not available on CRAN, it can be installed from the archive at https://cran.r-project.org/src/contrib/Archive/betaboost/.

Load packages and read data

```
library(tidyverse)
library(wavelets)
library(betaboost)
bpRange <- 81:336
oscillationRange <- 81:141

FLD <- readRDS("data/sWGS-master/fragment_lengths_deidentified_final.Rds") %>%
  as_tibble() %>%
  mutate(length = 1:n()) %>%
  filter(length %in% bpRange) %>%
  pivot_longer(-length, names_to = "Sample", values_to = "count") %>%
  # Compute the log cfDNA length distribution per sample.
  group_by(Sample) %>%
  mutate(freq.log = log(count) - log(sum(count))) %>%
  ungroup()
ddPCR_patient_cfDNA <- read_csv("data/sWGS-master/ddPCR_patient_cfDNA.csv")
ichorCNA_TC_estimates <- read_csv("data/sWGS-master/ichorCNA_TC_estimates.csv")
CRP_TC_estimates <- read_csv("data/sWGS-master/CRP_TC_estimates.csv")

dict <- ichorCNA_TC_estimates %>%
  left_join(CRP_TC_estimates, by = "Sample")
```

Detrend, Fourier and wavelet transform

For the Fourier transform, we regress out a second-degree polynomial in the [81, 141] bp interval, and transform the residuals. The wavelet transform is applied to the [81, 336] bp interval, using Daubechies

wavelet function of length 10.

```
FLD.detrend.tsfm <- FLD %>%
  group_by(Sample) %>%
  nest() %>%
  mutate(detrend = map(data, function(nested) {lm(freq.log ~ poly(length, 2),
                                                  data = nested,
                                                  subset = length %in%
                                                    oscillationRange)}),
         fourier = map(detrend, function(dtr) tibble(index = 1:length(dtr$residuals),
                                                     dft = fft(dtr$residuals))),
         wavelet = map(data, function(nested) {dwt(nested$freq.log,
                                                   filter = "d10")}),
         # Flatten the lists returned by `dwt`, reflecting different scales.
         wavelet.coefs.flat = map(wavelet, function(wave) {
           # Extract the wavelet coefficients (a list of different scales).
           attr(wave, "W") %>%
             # Tidy up and index the coefficients within scales.
             lapply(function(z) { as.data.frame(z) %>%
                 as_tibble() %>% mutate(coef = 1:n()) }) %>%
             # Merge scales into one tibble, where `Wcoef` stores coefficient
             # on scale x with index y as Wx_y.
             bind_rows(.id = "W") %>%
             rename(value = `V1`) %>%
             unite(Wcoef, W, coef, sep = "_")
         })) %>%
  ungroup()
```

Unnest and prepare features

Fourier coefficients

```
FLD.FFT.spread <- FLD.detrend.tsfm %>%
  select(Sample, fourier) %>%
  unnest(fourier) %>%
  # Index 1 is 0 (due to centring). Let's shift to zero-based indexing.
  filter(index %in% 2:31) %>%
  mutate(mod = Mod(dft),
         Fcoef = paste0("F", index - 1)) %>%
  select(Sample, Fcoef, mod) %>%
  spread(Fcoef, mod)
```

Wavelet coefficients

```
FLD.DWT.spread <- FLD.detrend.tsfm %>%
  select(Sample, wavelet.coefs.flat) %>%
  unnest(wavelet.coefs.flat) %>%
  spread(Wcoef, value)
```

Merge all features and split data for variable selection and parameter tuning

```
FFT.DWT <- FLD.FFT.spread %>%
  left_join(FLD.DWT.spread, by = c("Sample"))
```

```
DFT.DWT.dict <- FFT.DWT %>%
  left_join(dict, by = "Sample")

DFT.DWT.cohortB <- DFT.DWT.dict %>%
  # Keep patient samples without CRP TC but with non-zero ichorCNA TC.
  filter(str_detect(Sample, "^s_DS_fastcf"), is.na(`Estimated TC`), `ichorCNA TC` > 0) %>%
  rename(TC = `ichorCNA TC`) %>%
  select(TC, starts_with("F", ignore.case=F), starts_with("W", ignore.case=F))

DFT.DWT.cohortA <- DFT.DWT.dict %>%
  filter(str_detect(Sample, "^s_DS_fastcf"), !is.na(`Estimated TC`)) %>%
  mutate(TC = `Estimated TC`) %>%
  select(TC, starts_with("F", ignore.case=F), starts_with("W", ignore.case=F))

DFT.DWT.dilution <- FFT.DWT %>%
  filter(str_detect(Sample, "^Her2_dilution")) %>%
  left_join(ddPCR_patient_cfDNA, by = c("Sample" = "sample_name_rep"))
```

Gradient boosting and stability selection

```
# Fit boosted beta-regression model.
bboost.fit <- betaboost(TC ~ ., data = DFT.DWT.cohortB, iterations = 250)
# Perform stability selection.
stabsel_parameters(p = ncol(DFT.DWT.cohortB) - 1, PFER = 1, cutoff = 0.6,
                   assumption = "r-concave")
```

```
## Stability selection with r-concavity assumption
##
## Cutoff: 0.6; q: 15; PFER (*):  0.877
##    (*) or expected number of low selection probability variables
## PFER (specified upper bound):  1
## PFER corresponds to signif. level 0.00325 (without multiplicity adjustment)
```

```
set.seed(1)
bboost.stabsel <- stabsel(bboost.fit, PFER = 1, cutoff = 0.6,
                          assumption = "r-concave")
plot(bboost.stabsel, type = "paths")
```

The following features are selected based on cohort B:

```
bboost.stabsel$selected
```

```
##   F11 W1_35 W2_18 W3_20 W3_29  W4_8
##     4    89   169   236   245   270
```

Fit final boosting model

```
bboost.fit.CRP <- betaboost(TC ~ .,
                            data = DFT.DWT.cohortA %>%
                              select(TC, names(bboost.stabsel$selected) %>%
                                       setdiff("(Intercept)")),
                            iterations = 250)
cvr.CRP <- cvrisk(bboost.fit.CRP)
plot(cvr.CRP)
```

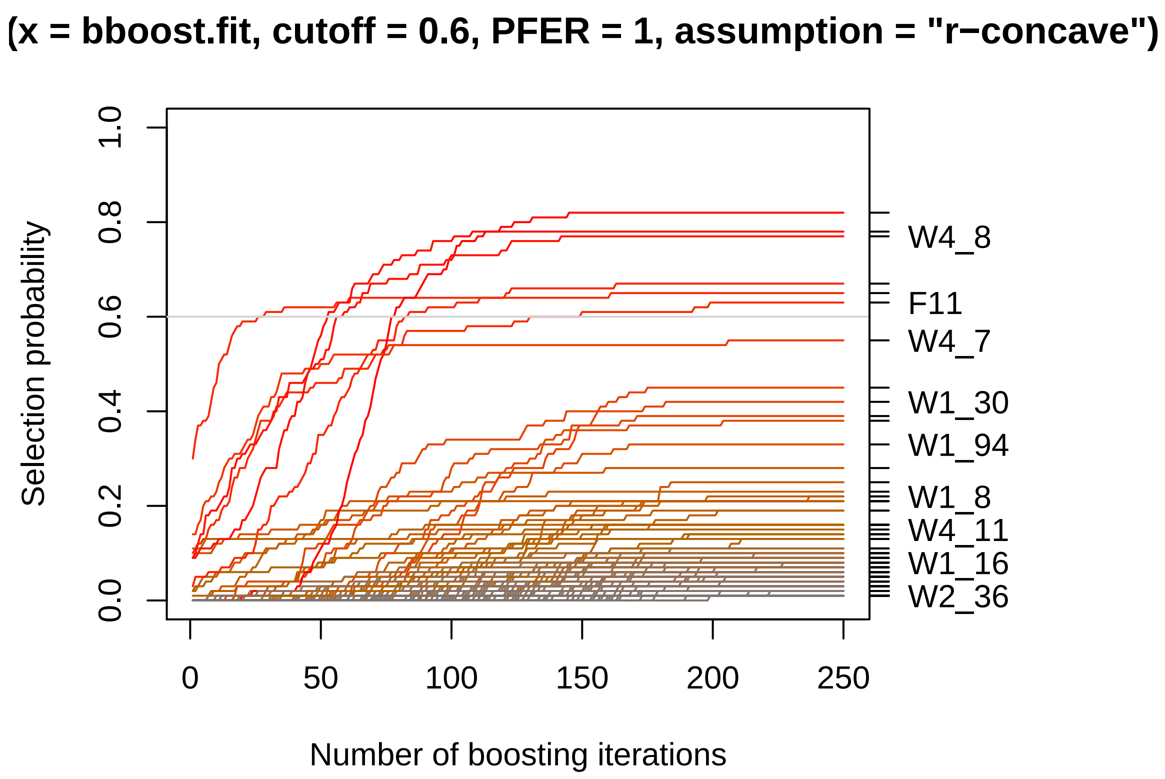

Figure S4: Stability paths during boosting in cohort B.

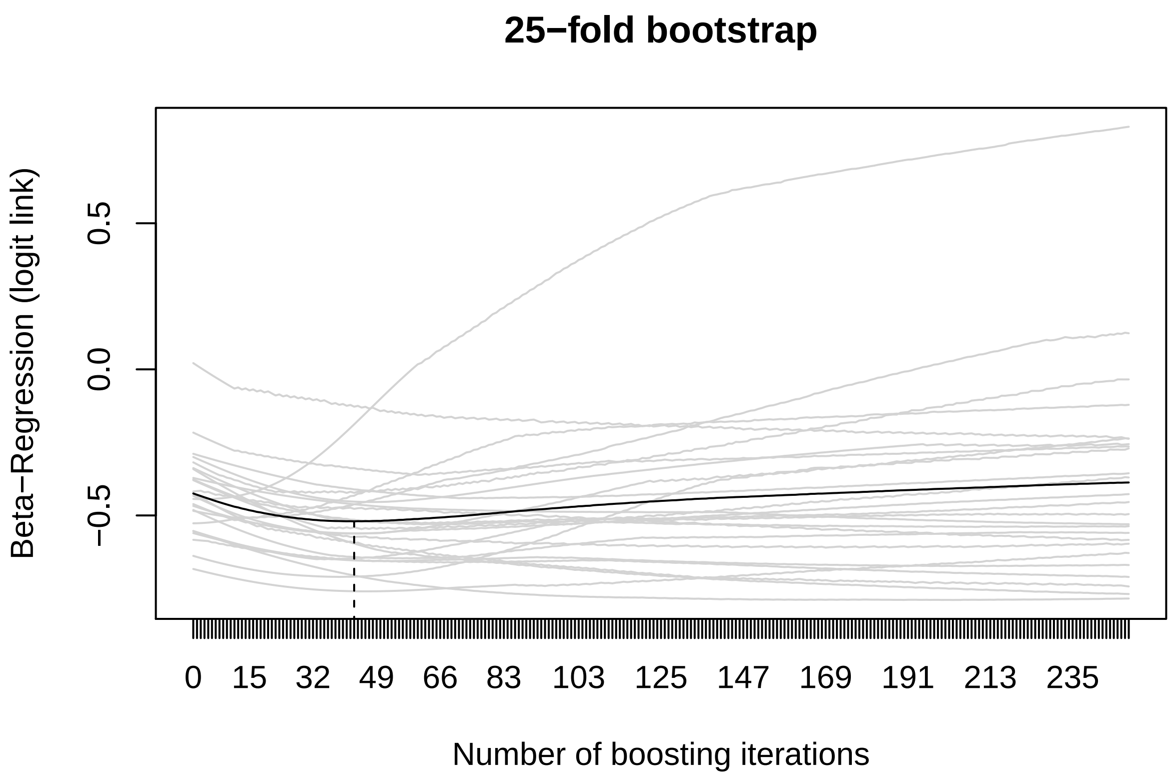

Figure S5: Cross-validation risk during boosting in cohort A.

```
mstop(bboost.fit.CRP) <- mstop(cvr.CRP)
```

Test set prediction

```
gg.her2.TCprediction <- tibble(
  `predicted tumour content` = predict(bboost.fit.CRP,
                                       newdata = DFT.DWT.dilution,
                                       type = "response")[,1],
  `patient-derived cfDNA, %` = DFT.DWT.dilution$`patient-derived cfDNA, %`)

gg.her2.TCprediction %>%
  ggplot(aes(`patient-derived cfDNA, %`, `predicted tumour content`)) +
  geom_point() +
  geom_smooth(method = "lm") +
  geom_smooth(method = "lm", formula = y ~ 0 + x, se = F, lty = "dashed",
              colour = "black", size = 0.5)
```

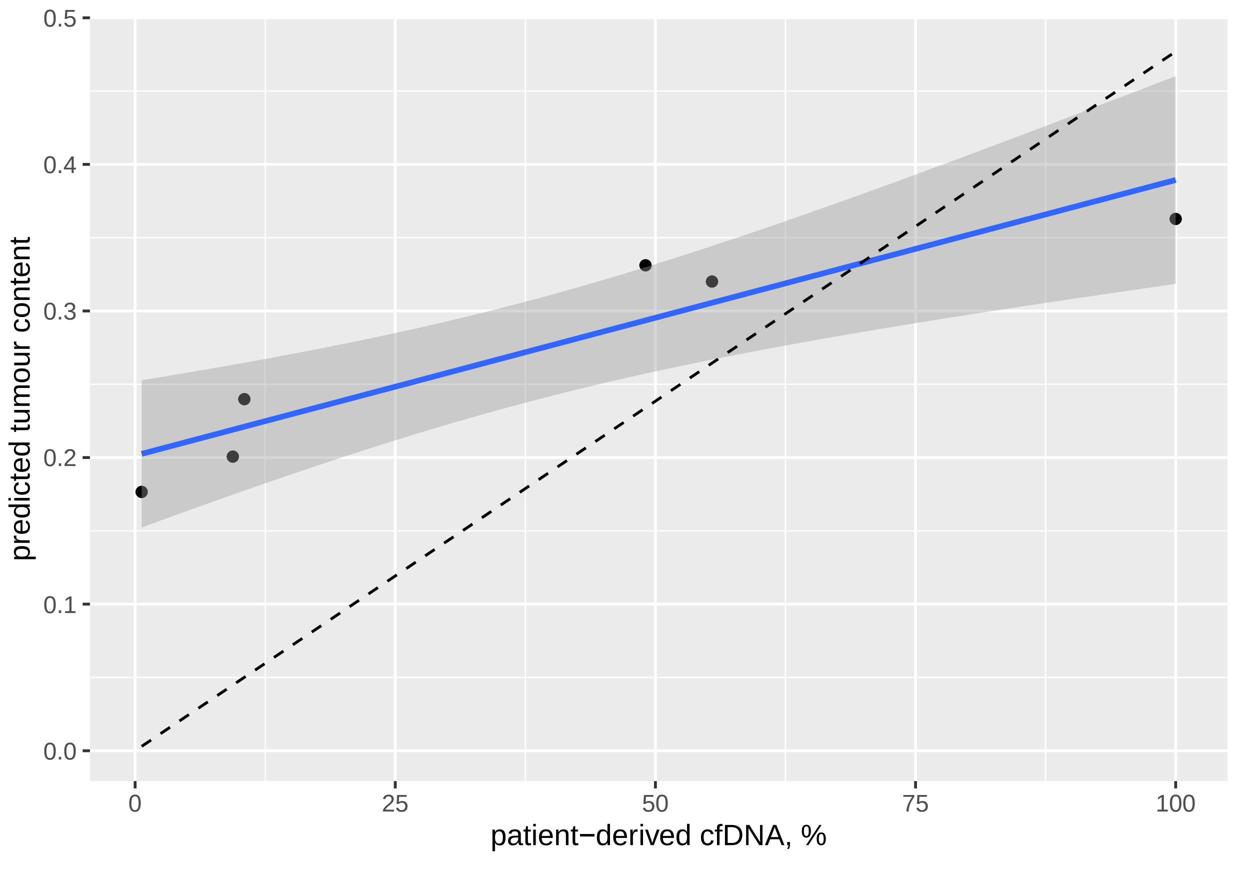

Figure S6: Predicted tumour content vs dilution. This corresponds to the main Figure 2b.

The summary statistics of the linear fits, with an without an intercept terms, are as follows.

```
lm(`predicted tumour content` ~ `patient-derived cfDNA, %`,
   data = gg.her2.TCprediction) %>%
  summary()
```

```
##
```

```
## Call:
## lm(formula = `predicted tumour content` ~ `patient-derived cfDNA, %`,
##     data = gg.her2.TCprediction)
##
## Residuals:
##        1        2        3        4        5        6
## -0.02595  0.01876 -0.01835  0.03760  0.01451 -0.02657
##
## Coefficients:
##                            Estimate Std. Error t value Pr(>|t|)
## (Intercept)                0.201340   0.018250  11.032 0.000384 ***
## `patient-derived cfDNA, %` 0.001880   0.000357   5.266 0.006229 **
## ---
## Signif. codes:  0 '***' 0.001 '**' 0.01 '*' 0.05 '.' 0.1 ' ' 1
##
## Residual standard error: 0.03038 on 4 degrees of freedom
## Multiple R-squared:  0.8739, Adjusted R-squared:  0.8424
## F-statistic: 27.73 on 1 and 4 DF,  p-value: 0.006229
```

```
lm(`predicted tumour content` ~ 0 + `patient-derived cfDNA, %`,
   data = gg.her2.TCprediction) %>%
  summary()
```

```
##
## Call:
## lm(formula = `predicted tumour content` ~ 0 + `patient-derived cfDNA, %`,
##     data = gg.her2.TCprediction)
##
## Residuals:
##        1        2        3        4        5        6
##  0.17358  0.18977  0.15586  0.09721  0.05566 -0.11418
##
## Coefficients:
##                            Estimate Std. Error t value Pr(>|t|)
## `patient-derived cfDNA, %` 0.004770   0.001217   3.921   0.0112 *
## ---
## Signif. codes:  0 '***' 0.001 '**' 0.01 '*' 0.05 '.' 0.1 ' ' 1
##
## Residual standard error: 0.1523 on 5 degrees of freedom
## Multiple R-squared:  0.7546, Adjusted R-squared:  0.7055
## F-statistic: 15.37 on 1 and 5 DF,  p-value: 0.01118
```

Session information

```
sessionInfo()
```

```
## R version 4.0.5 (2021-03-31)
## Platform: x86_64-apple-darwin17.0 (64-bit)
## Running under: macOS Big Sur 10.16
##
## Matrix products: default
## BLAS:   /Library/Frameworks/R.framework/Versions/4.0/Resources/lib/libRblas.dylib
## LAPACK: /Library/Frameworks/R.framework/Versions/4.0/Resources/lib/libRlapack.dylib
##
## locale:
```

```
## [1] en_US.UTF-8/en_US.UTF-8/en_US.UTF-8/C/en_US.UTF-8/en_US.UTF-8
##
## attached base packages:
## [1] parallel  stats     graphics  grDevices utils     datasets  methods
## [8] base
##
## other attached packages:
##  [1] betaboost_1.0.1   gamboostLSS_2.0-5 mboost_2.9-5      stabs_0.6-4
##  [5] wavelets_0.3-0.2  lubridate_1.9.2   forcats_1.0.0     stringr_1.5.0
##  [9] dplyr_1.1.2       purrr_1.0.1       readr_2.1.4       tidyr_1.3.0
## [13] tibble_3.2.1      ggplot2_3.4.2     tidyverse_2.0.0
##
## loaded via a namespace (and not attached):
##  [1] mvtnorm_1.1-3     lattice_0.20-41   digest_0.6.33     utf8_1.2.3
##  [5] R6_2.5.1          nnls_1.4          evaluate_0.21     highr_0.10
##  [9] pillar_1.9.0      rlang_1.1.1       rstudioapi_0.15.0 rpart_4.1-15
## [13] Matrix_1.3-2      partykit_1.2-15   rmarkdown_2.23    labeling_0.4.2
## [17] splines_4.0.5     bit_4.0.5         munsell_0.5.0     compiler_4.0.5
## [21] xfun_0.39         pkgconfig_2.0.3   libcoin_1.0-9     mgcv_1.8-34
## [25] htmltools_0.5.5   tidyselect_1.2.0  quadprog_1.5-8    fansi_1.0.4
## [29] crayon_1.5.2      tzdb_0.4.0        withr_2.5.0       grid_4.0.5
## [33] nlme_3.1-152      gtable_0.3.3      lifecycle_1.0.3   magrittr_2.0.3
## [37] scales_1.2.1      cli_3.6.1         stringi_1.7.12    vroom_1.6.3
## [41] farver_2.1.1      generics_0.1.3    vctrs_0.6.3       Formula_1.2-5
## [45] tools_4.0.5       bit64_4.0.5       glue_1.6.2        hms_1.1.3
## [49] fastmap_1.1.1     survival_3.2-10   yaml_2.3.7        timechange_0.2.0
## [53] colorspace_2.1-0  inum_1.0-4        knitr_1.43
```
